# Editorial: Advances in contemplative sciences

**DOI:** 10.3389/fpsyg.2025.1545836

**Published:** 2025-01-21

**Authors:** Javier García-Campayo, Jesús Montero-Marin, Roberto Aristegui

**Affiliations:** ^1^Psychiatry, University of Zaragoza, Zaragoz, Spain; ^2^Psychology, University of Oxford, Oxford, United Kingdom; ^3^Escuela de Psicología, Universidad Adolfo Ibáñez, Peñalolén, Chile

**Keywords:** mindfulness, meditation, advances, research, contemplative

In his 1930 book “Civilization and Its Discontents,” Freud advocated a new and secular science of psychotherapy as a pragmatic alternative to the traditional healing techniques offered by religions. Since then, Western psychology/psychiatry has operated separately from religion (Neeleman and Persaud, 1995). However, in recent years, robust research has confirmed the potential of qualities such as love, compassion, and altruism for therapeutic use and wellbeing (Lutz et al., 2008; Amihai and Kozhevnikov, 2014). For this reason, conventional psychology is moving toward contemplative science, which has always been considered nonscientific knowledge.

Davidson and Begley (2013) coined the term “contemplative neuroscience,” which describes the emerging field arising at the intersection of meditation research and neuroscience. It includes any kind of meditation, yoga, mantra recitation, intensive breathing, and healing movement (Loizzo, 2009). This new science fulfills the dream of visionary pioneers in psychiatry and psychology, such as Carl Gustav Jung and Ken Wilber, and is connected to some aspects of transpersonal psychology. Several universities across the world have developed courses on or departments of contemplative sciences or similar disciplines, examples of which include Brown University (Providence, Rhode Island), the University of San Diego (San Diego, California), Naropa University (Boulder, Colorado) and the University of Mary Washington (Fredericksburg, Virginia), in the United States, and the University of Ottawa, in Canada.

In recognition of this Research Topic, we have edited a dedicated Research Topic on Frontiers in Psychology. It comprises nine papers, seven clinical or experimental, and two theoretical ones that tackle different aspects of meditation. With regard to clinical studies, the study by Buric et al. compares three groups of participants (sensory priming, semantic priming, and control group). The intervention was administered prior to a guided self-compassion meditation, and three variables were assessed: state self-compassion, self-criticism, and positive and negative affect. Despite being underpowered, the study suggests that neither semantic nor sensory priming improves any of the outcomes.

The study by McDonald et al. assesses the efficacy of online mindfulness in reducing suspiciousness/paranoia in individuals with high positive schizotypy. A 40-day mindfulness-based intervention was compared to an active control condition using reflective journaling. The feasibility criteria were excellent in the intervention group: 100% retention, a completion rate of 91%, and high acceptance. Mindfulness produced no effect on trait paranoia but reduced state paranoia with a medium-to-large effect.

The study by Neri et al. assesses the neural correlates of concentrative and analytical meditation, the two most important forms of Tibetan meditation. At the Tibetan University of Sera-Jey in India, 23 meditators underwent an ecological EEG acquisition. Concentrative meditation elicited more numerous and marked changes than analytical meditation, consisting of an increase in the theta, alpha, and beta frequency ranges.

The paper by Borghi et al. analyzes the relationship between daily perceived stress and mindfulness, specifically, and the four facets of mindfulness: observing, describing, nonjudging, and nonreacting. Bidirectional cross-lagged associations were investigated using the random-intercept cross-lagged panel model. The results challenge prior results regarding mindfulness as a protective factor against daily stress. With the exception of nonreacting, mindfulness was either positively associated with perceived stress, or else perceived stress appeared to interfere with the ability to stay mindful in daily life.

The study by McGee et al. paper assesses collective labyrinth walking as an integrative contemplative practice, particularly at times of distress, such as during pandemics. The study was developed at the height of the COVID-19 pandemic by 416 participants from 19 countries. Three predominant themes emerged in the qualitative study: (1) A sense of connectedness between the participants, (2) Qualities associated with “transcendent” experiences (boundless, ultimacy, transcendence), and (3) Compassionate action.

The paper by Price et al. studies the effect of a mindfulness-based protocol whose aim was to improve mind wandering by reviewing five longitudinal studies conducted in different organizational settings. A meta-analysis confirmed differences between the mindfulness and control groups between baseline and 4-month follow-up in mind wandering and meta-awareness, with low-to-intermediate effects.

The study by Smith et al. reports on the efficacy of mindfulness and yoga compared to usual training in health, pain, and injury on a sample of US Army trainees. The objective outcomes were injury-related medical encounters and the number of diagnoses. After the intervention, the trainees showed better health, sleep, and mood, less stress and impact of pain on training, and fewer diagnoses. Differences arose at 3 weeks.

In addition, the following two theoretical studies are included. The first is an introspective paper by Sparby et al. on six individuals performing different attentional tasks over a month. The authors discuss whether our inability to sustain attention implies we are not free. It also reflects on the role of introspection as a method in contemplative sciences. Finally, the paper by Garcia-Campayo et al. discusses the need for personalized meditation according to meditators' characteristics. It reviews several personality classifications—two from the Buddhist tradition and the Enneagram—that could be useful to predict which meditation better fits specific personality traits or characteristics.

In summary, this Research Topic aims to present some of the new research areas currently in progress within the domain of contemplative sciences.
